# Job Satisfaction and Negative Coping Style Affect the Relationship between Transition Shock and Intent to Stay among Newly Graduated Nurses during the COVID-19 Pandemic

**DOI:** 10.1155/2023/4286004

**Published:** 2023-05-04

**Authors:** Yaqi Zhu, Wenjuan Tang, Yuanyuan Zhang, Mengyao Li, Weiyi Zhu, Yaqing Zhang

**Affiliations:** ^1^Shanghai Jiao Tong University, School of Nursing, Shanghai, China; ^2^Shanghai Children's Hospital, Shanghai Jiao Tong University, School of Medicine, Shanghai, China; ^3^Ruijin Hospital, Shanghai Jiao Tong University, School of Medicine, Shanghai, China

## Abstract

**Aim:**

The study is aimed at exploring the relationship between newly graduated nurses' transition shock, negative coping, job satisfaction, and intent to stay during the time of COVID-19.

**Background:**

The shortage of nurses is a global dilemma aggravated by the COVID-19 pandemic. It has been a hot topic in recent years to help newly graduated nurses transition smoothly. Transition shock is one of the essential indicators to describe the transition state of newly graduated nurses, which has a far-reaching impact on the intention of newly graduated nurses to stay in their posts. However, few studies have studied the mechanism behind this relationship, which may affect the effectiveness of retention strategies.

**Methods:**

A descriptive cross-sectional study was conducted from July to August 2021 in 31 tertiary hospitals in Shanghai, China. Participants comprised a convenience sample of 759 newly graduated nurses for surveys. Structural equation models were used to examine the study's hypothetical model.

**Results:**

The results showed that transition shock had a significant direct effect on job satisfaction (*b* = −0.412, *p* < 0.001) and intent to stay (*b* = −0.145, *p* < 0.001). Job satisfaction had a significant direct effect on intent to stay (*b* = 0.702, *p* < 0.001). The indirect effect of transition shock on intent to stay through job satisfaction was statistically significant (*b* = −0.289), the 95% C.I. was (−0.493, −0.357), and the proportion of mediating effect to total effect was 66.59%. Moreover, the moderated mediation analysis showed that the interaction effect of transition shock and negative coping style on job satisfaction was significant (*b* = −0.082, *p* < 0.001).

**Conclusion:**

This study revealed the impact of transition shock on intent to stay of newly graduated nurses during the time of COVID-19, and found that job satisfaction played a mediating role and negative coping played a moderating role. These findings are of great significance for nursing managers to take measures to improve the intention of newly graduated nurses to stay. *Implication for Nursing Management*. The level of transition shock is an important indicator reflecting the transition state of newly graduated nurses, and can further predict the job satisfaction and intention of newly graduated nurses to stay. Therefore, nursing managers should pay attention to taking corresponding measures to reduce the level of transition shock of newly graduated nurses.

## 1. Introduction

For the recent decades, the shortage of nurses has been a global problem that needs to be solved urgently [1]. The nursing deficit will reach 6 million by 2030, according to the World Health Organization (WHO) [2]. In recent years, due to the aging of the population, the demand for nurses in various countries has been rising [3]. However, the impact of the COVID-19 pandemic on the mental health of nurses may aggravate the nursing shortage in terms of burnout and anxiety caused by pandemic-related factors like high infection levels and ever-increasing workloads [4, 5]. Therefore, joining newly graduated nurses in the healthcare becomes especially important [6, 7]. Nevertheless, the turnover rate of newly graduated nurses is an increasingly severe problem [8]. The studies carried out around the world showed that a great number of newly graduated nurses have the intent to leave the post within the first year of employment [9–11]. A Chinese study found that up to 74.4% of newly graduated nurses have the intent to leave the organization because of the stress in the transition period [12]. Hence, it has become a hot research topic in recent years to adopt the best strategy to help newly graduated nurses pass through the transition period smoothly, so as to improve the retention rate of newly graduated nurses in healthcare settings [13, 14].

It should not be overlooked that newly graduated nurses would go through a difficult time when they first enter work after graduation [15]. The transition shock of newly graduated nurses is defined as the disturbing or discordant experiences at the initial stage of role adaptation caused by the significant difference between theoretical and practical expectations and reality [15]. Duchscher explained the transition shock of newly graduated nurses from four perspectives: emotional, sociodevelopmental and cultural, physical, and intellectual factors [15]. If not adequately supported, nurses may be trapped in constant self-doubt and fear at this stage. Studies have shown that transition shock exists not only in newly graduated nurses but also in junior nurses with ≤5 years of service [16]. The excessive transition shock of newly graduated nurses may lead to a decrease in their professional quality of life [17] and even affect the safety of patients [18, 19]. The COVID-19 pandemic has made it more difficult for newly graduated nurses to overcome transition shock [20]. In addition to the inherent problems in the transition period, the prevalence of COVID-19 has also caused newly graduated nurses to face the impact of increased workload, reduced on-the-job training, and increased risk of contracting COVID-19 on their physical and mental health [21]. In China, newly graduated nurses are facing a similar situation. In view of the fact that some experienced nurses needed to go out to support mobile cabin hospital during the COVID-19 period, the newly graduated nurses faced greater work pressure [22]. Meanwhile, the COVID-19 pandemic also leads to inadequate preparations for entering clinical work, such as interruption of clinical practice, change in the form of classroom education, economic pressures, and increased concerns about delayed graduation [23]. All the changes mentioned above will cause increased negative emotions in newly graduated nurses in the transition period, which will evolve into a stronger transition shock [20, 24].

### 1.1. Transition Shock and Intent to Stay, Job Satisfaction

The definition of intent to stay of nurses refers to their views on the likelihood of staying in their current post [25]. The higher the nurses' intent to stay, the lower the burnout level of nurses, and the lower the mortality rate and the incidence of adverse outcomes of patients [26]. Moreover, retaining newly graduated nurses is beneficial to avoid wasting the training cost invested by hospitals in the early stage [27]. Studies have shown that good transition programs can help improve the retention rate of newly graduated nurses [27, 28]; although some qualitative studies have shown a correlation between newly graduated nurses' satisfaction with the transition and their intent to stay [29, 30].

Multiple studies found that newly graduated nurses' job satisfaction and their intent to stay are closely intertwined [31, 32]. The more satisfied newly graduated nurses are with the job, the more likely they will stay at their current post [31–33]. Although Kim and Yeo found that the transition shock of newly graduated nurses might be associated with their job satisfaction, the mechanism remains unclear [34].

### 1.2. Transition Shock and Negative Coping

When experiencing transition shock, newly graduated nurses usually have negative emotions such as anxiety and depression [35, 36]. Therefore, they may adopt negative coping strategies to avoid unpleasant experiences [37, 38]. Coping styles are defined as the ideas or behaviors adopted by individuals to cope with adversity and stress [39]. Coping styles can be divided into negative coping and positive coping [40]. Negative coping refers to solving problems by avoiding or withdrawing, while positive coping refers to solving issues directly and rationally [40]. However, there is an asymmetry between negative coping and positive coping [41]. Compared with positive coping, negative coping has a more significant functional influence and is more susceptible to changes due to external intervention [38, 41, 42]. A study showed that negative coping could moderate the relationship between the self-efficacy of medical staff and the post-traumatic stress response caused by the COVID-19 pandemics [43]. Nevertheless, adopting negative coping will not change its destructive or threatening nature [44]. Instead, it just temporarily maintains personal feelings in a relatively good state [44].

### 1.3. Literature Summary

Considering the difficulties faced by newly graduated nurses in the transition during the COVID-19 period, the rigorous research is needed to identify the appropriate strategies to help them successfully survive the transition shock and improve their intent to stay. However, though previous studies have indicated the relation between newly graduated nurses' transition shock and their intent to stay [29, 30], the roles of negative coping and job satisfaction in the relation between the two have not been analyzed in depth in any literature, especially in the particular period of the COVID-19 pandemic. To this end, the major goal of the present study was to investigate the associations among transition shock, negative coping, job satisfaction, and intent to stay in a sample of Chinese newly graduated nurses during the COVID-19 period.

### 1.4. Theoretical Foundation and Research Hypotheses

The theoretical framework employed in this study based on the theoretical model of job retention, the theory of organizational socialization, and the conservation of resources (COR) theory.

According to the theoretical model of job retention, job satisfaction is directly related to the intent to stay of nurses, and their job satisfaction and personal characteristics indirectly affect their choice of retention through their intent to stay [45]. According to Ellenbecker [45], retention is defined as the extent to which nurses stay in their present jobs, and job satisfaction is defined as a positive affective orientation toward employment. The model effectively explains the relationship between job characteristics, job satisfaction, intent to stay, and retention rate.

Besides, organizational socialization, or onboarding, is defined as a process in which new employees become internal personnel of the organization [46]. Bauer and Erdogan [46] summarized the factors affecting the organizational socialization of new employees into the following three aspects: new employee characteristics, new employee behavior, and organizational effort. In the face of a new environment, a proactive personality and supportive organizational strategies help new employees adapt to the new organization [46]. After experiencing the role conflict of transition, newly graduated nurses finally achieve the results of organizational socialization through adjustment and adaptation with the help of joint effort between individuals and organizations [47]. The results of organizational socialization have been mainly measured by job satisfaction, retention, or turnover [47].

COR theory holds that individuals would always try to protect and preserve the resources they own and value [48]. Resources are divided into internal resources and external resources [49]. Internal resources include physical and emotional energy, while external resources include energy that an individual hopes to obtain from the outside world [49]. Newly graduated nurses may experience a loss of these resources when they experience transition shock [15]. When resources are lost, individuals often take the way of making up for or avoiding resource loss to deal with it [50]. In the process of avoiding further loss of resources, individuals may take the initiative to reduce their self-expectations of the role and adopt a negative coping style of feedback avoidance [51].

**Figure 1:**
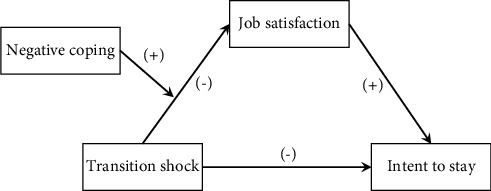
The theoretical model of this study.

Therefore, on the abovementioned theoretical basis, the following hypothesis was proposed, and the theoretical model of this study was built, as shown in Figure 1:

  (H1). Transitional shock has a direct negative impact on job satisfaction
  (H2). Transitional shock has a direct negative impact on intent to stay
  (H3). Job satisfaction has a direct positive impact on intent to stay
  (H4). Job satisfaction plays a mediating role between transition shock and the intent to stay
  (H5). Negative coping plays a moderating role between transition shock and job satisfaction

## 2. Methods

### 2.1. Design

A descriptive cross-sectional survey design was adopted in this study followed by the STROBE Statement: guidelines for reporting observational studies (see the supplementary file (available here)).

### 2.2. Settings and Participants

PASS 2021 was adopted for the calculation of sample size in the current study. According to the prevalence of retention intention in Chinese nurses (standard deviation (SD) = 0.67) [25], a smallest sample size of 690 newly graduated nurses was required, with power of 0.95 and alpha of 0.05. Assuming an attrition rate of 20%, a total of 828 newly graduated nurses were invited.

This study was conducted in 31 tertiary hospitals in Shanghai, China, which were selected through convenience sampling. The inclusion criteria in this study were newly graduated nurses with less than one year of work experience. The exclusion criteria were newly graduated nurses who did not work at the surveyed hospitals during the survey period (i.e., sick leave, participating in continuing education programs). The response rate was 91.7% (*n* = 759).

### 2.3. Data Collection

Data collection was undertaken from July to August 2021. Dr. Tang presented the project to the director of the nursing department in the hospitals and distributed the questionnaire electronically. Then, the nurses who meet the inclusion criteria received the questionnaire electronically to complete. The first page of the electronic questionnaire is the informed consent. Before entering the filling page, participants need to read the informed consent, including the research purpose, and rights and obligations, and click “Confirm to participate in this study” before entering the formal questionnaire filling page. All the participants were assured that their participation in the study was strictly voluntary and anonymous. After that, the first author collected the answers via the questionnaire database.

### 2.4. Measures

The questionnaire we used consists of four parts: the demographic datasheet, The Transition Shock of Newly Graduated Nurses Scale [52], The Trait Coping Style Questionnaire [53], and Nurse Job Satisfaction Scale [54].

The demographic datasheet comprises five questions related to age, gender, level of education, marital status, and native place.

The Transition Shock of Newly Graduated Nurses Scale (TSS-NGNs) was developed by Xue et al. [52]. Each item was rated on a 5-point Likert scale (ranging from 1 = “strongly disagree” to 5 = “strongly agree”). This scale consists of 27 items covering four dimensions entitled: “emotional” (8 items, e.g., “Sometimes I feel lonely and inferior”), “sociodevelopmental and cultural” (8 items, e.g., “I do not often express my opinions in the department where I work.”), “physical” (6 items, e.g., “My skin is getting rough and dry.”), and intellectual' (5 items, e.g., “I do not know how to effectively cope with the doubts of patients and their family.”). Cronbach's alpha coefficients ranging from 0.873–0.920 for the four dimensions and 0.962 for the total scale are explained in this study.

To assess the degree of negative coping, we adopted the 10-item negative coping subscale of Trait Coping Style Questionnaire (TCSQ) [53]. Each item was rated on a 5-point Likert scale (ranging from 1 = “strongly disagree” to 5 = “strongly agree,” e.g., “Unpleasant things can easily cause my mood swings”). The scores indicate the tendency to use negative coping, with higher scores representing a greater tendency to use negative coping. The negative coping subscale has shown good internal consistency and test-retest reliability [53]. In the current study, Cronbach's alpha for the negative coping subscale of TCSQ was 0.895.

Nurse Job Satisfaction Scale (NJSS) was used to assess the degree of job satisfaction of newly graduated nurses [55]. The scale is composed of 38 items representing eight dimensions. Each item was rated on a 5-point Likert scale (ranging from 1 = “strongly disagree” to 5 = “strongly agree,” e.g., “The doctor and I worked very well together”). In the current study, Cronbach's alpha was 0.954 for the total scale.

To assess the degree of intent to stay, we adopted the 6-item Nurses' Intent to Stay Scale (NITSS) [54]. Each item was rated on a 5-point Likert scale (ranging from 1 = “strongly disagree” to 5 = “strongly agree,” e.g., “I never considered leaving the nursing post.”). The second, third, and sixth items in the scale are negative items, the results of which are counted in reverse (e.g., “I would consider leaving my nursing position if other job opportunities (other than nursing) become available”). The scores indicate the degree of intent to stay, with higher scores representing a greater tendency to stay in the current position. The Cronbach's alpha was 0.77 and the content validity index was 0.97.

### 2.5. Data Analysis

Mplus7.0 was used to analyze the data. Descriptive analyses were performed using frequency, percentage, mean, and standard deviation. Pearson's correlation coefficient was used to examine the associations between the study variables. Structural equation model, which consists of a set of multivariate techniques that allow the researcher to test theory-guided hypotheses with clearly confirmatory ends [56], was utilized to test the hypothetical model of the current study. The maximum likelihood method was used to estimate the structural equation model, and the fitting of the structural model was verified by chi-square (*χ*^2^)/degrees of freedom ratio (d*f*), standardized root mean square residual (SRMR), root mean square error of approximation (RMSEA), comparative fit index (CFI), and Tucker–Lewis index (TLI). The *χ*^2^/d*f* ≤ 5, TLI and CFI ≥0.90, and SRMR and RMSEA ≤0.08 were a reasonable-fit [57]. Confirmatory factor analyses were performed to ensure the validity of the study construct. The nonparametric bootstrap was used for mediator testing, sampling was repeated 5000 times. Bootstrap and simple slope method were used to test the moderating effect. The *p* value was two-tailed, with values below 0.05 showing statistical significance.

### 2.6. Ethical Consideration

The participants were free to fill out the questionnaire or quit the study. In addition, data collection in this study was conducted on an anonymous basis, and the answers of participants are guaranteed to be used only in this study. Therefore, this study involved no unethical conduct or human clinical trial and would bring no adverse health consequences for the participants, physically or mentally.

## 3. Results

### 3.1. Participants Characteristics

The participants consisted of 700 (92.2%) women and 59 (7.8%) men. 75.0% of nurses were under 22 years and 25% were over 23 years. In the current study, 502 (66.1%) nurses had college degrees, 238 (31.4%) nurses had bachelor's degrees, and 19 (2.5%) nurses had master's degrees or above. Most nurses (73.4%) were single, whereas 24.9% of nurses had boyfriends or girlfriends and 1.7% of nurses were married.

### 3.2. Reliability and Validity

Reliability was analyzed to verify the measures used. The results of the study indicated that Cronbach's alpha values ranged from 0.829 to 0.962 for all scales, showing a high degree of internal consistency of each scale [58]. Confirmatory factor analysis showed that the four-factor model performed the best (*χ*^2^/d*f* = 3.076, RMSEA = 0.052, SRMR = 0.041, CFI = 0.940, and TLI = 0.934) compared to other models (see Table 1), which proved the variables had good construct validity and discriminant validity.

### 3.3. Correlation Analysis

The values of the mean and standard deviation of the variables and the correlation matrices for the variables are presented in Supplementary Material Table 1. The average score of transition shock was (2.991 ± 0.813), job satisfaction was (3.280 ± 0.501), intent to stay was (3.433 ± 0.731), and negative coping was (2.830 ± 0.708).

Pearson's correlation analysis indicated that transition shock was positively associated with negative coping (*r* = 0.623, *p* < 0.01), and negatively associated with job satisfaction (*r* = −0.547, *p* < 0.01) and intent to stay (*r* = −0.445, *p* < 0.01). Furthermore, negative coping was negatively associated with job satisfaction (*r* = −0.420, *p* < 0.01) and intent to stay (*r* = −0.313, *p* < 0.01). Finally, job satisfaction was significantly and positively associated with intent to stay (*r* = 0.638, *p* < 0.01).

### 3.4. Hypothesis Test

#### 3.4.1. Mediation Effects of Job Satisfaction

According to the results of path analysis (see Table 2 and Supplementary Material Figure 1), transition shock negatively predicted job satisfaction (*b* = −0.412, *p* < 0.001), and H1 was supported. Moreover, transition shock negatively predicted intent to stay (*b* = −0.145, *p* < 0.001), and H2 was supported. Finally, job satisfaction positively predicted intent to stay (*b* = 0.702, *p* < 0.001), and H3 was supported.

We adopted the Bootstrap method to test the significance of mediating path. As shown in Table 3, the indirect effect of transition shock on intent to stay through job satisfaction was statistically significant (*b* = −0.289), the 95% C.I. was (−0.493, −0.357) which did not contain 0. Furthermore, transition shock also had a direct effect on intent to stay (*b* = −0.145), the 95% C.I. was (−0.206, −0.067) which did not contain 0. Thus, job satisfaction partially mediated the effect of transition shock on intent to stay, and the proportion of mediating effect to total effect is 66.59%. Hence, H4 was supported.

#### 3.4.2. Moderating Effects of Negative Coping Style

The moderated mediation analysis (see Table 4) showed that the interaction effect of transition shock and negative coping style on job satisfaction was significant (*b* = −0.082, *p* < 0.001). The results in Table 4 indicated that the relationship between transition shock and job satisfaction was moderated by negative coping style, which means that H5 was supported.

**Figure 2:**
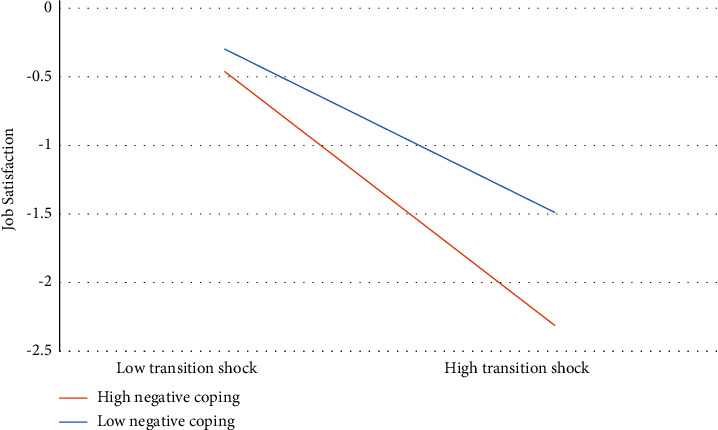
Moderating effect of negative coping.

The results of the simple slope test (see Figure 2 and Supplementary Material Table 2) indicated that, when the negative coping style was high, transition shock negatively predicted job satisfaction (*b* = −0.463, *p* < 0.001, bias-corrected confidence interval (BCCI) (−0.565, −0.376)). Moreover, when the negative coping style was middle, transition shock still negatively predicted job satisfaction (*b* = −0.380, *p* < 0.001, BCCI (−0.467, −0.303)), but the association was lower. Finally, when the negative coping style was low, the association between transition shock and job satisfaction was significant but the lowest (*b* = −0.298, *p* < 0.001, BCCI (−0.385, −0.216)). This result indicated that negative coping style regulated the relationship between transition shock and job satisfaction.

## 4. Discussion

The objective of this study was to explore the relationship between newly graduated nurses' transition shock, negative coping, job satisfaction, and intent to stay against the background of the COVID-19 pandemic. Firstly, it proved that transition shock of newly graduated nurses had a significant negative impact on their job satisfaction and intent to stay. Moreover, the results of this study showed that job satisfaction was partly mediated between the transition shock and the intent to stay. Finally, it was found that negative coping played a moderating role in the intermediary mechanism.

### 4.1. The Mediation of Job Satisfaction in the Relationship between Transition Shock and Intent to Stay

The results of this study showed that newly graduated nurses' transition shock was significantly negatively correlated to their intent to stay, which supported the results of previous studies [29, 59]. According to previous studies, the attrition rate of nurses was caused by the lack of confidence and the support available in the first few months of practice [28]. According to the transition theory of Duchscher and Windey, newly graduated nurses' transition period includes the doing, being, and knowing stages [60]. Their transition shock occurred in the first stage, the doing stage [60]. In addition, for millennial generation nurses, the main factors that made them stay at their posts were the alignment of organizational values, good coworker relationships, being recognized as a respected and valuable member of the team, and cutting-edge technology, such as resources and information easily accessible online [13]. These factors were exactly the important components in the transition shock faced by newly graduated nurses [15]. Therefore, in order to improve newly graduated nurses' retention rate, adopting appropriate measures to reduce the level of newly graduated nurses' transition shock was a critical point at the present time.

The results of this study showed that newly graduated nurses' transition shock was significantly negatively correlated to their job satisfaction, which confirmed our hypothesis and was consistent with the previous study [18]. Meanwhile, the results of this study also showed that job satisfaction mediated between the transition shock and the intent to stay. The relationship between job satisfaction and the intent to stay was repeatedly proven [31]. According to the transition shock theory of Duchscher, newly graduated nurses might be unable to complete the work on time or balance their work and life due to a lack of knowledge and unfamiliarity with the environment at the early stage of clinical work, which was one of the sources of the transition shock experienced by newly graduated nurses [15]. Previous studies have confirmed that the problem of balancing work and life might affect employees' mental health and job satisfaction and their decision to stay in their organization [61]. In addition, other components of newly graduated nurses' transition shock, such as their relationship with colleagues, also had a profound impact on their job satisfaction [62].

### 4.2. The Moderation of Negative Coping in the Relationship between Transition Shock and Job Satisfaction

Furthermore, it was found in this study that negative coping moderated the relation between transition shock and job satisfaction. The newly graduated nurses who adopted high negative coping could better predict job satisfaction than those who adopted low negative coping, which meant that high negative coping aggravated the negative prediction of transition shock on job satisfaction. Negative coping was deemed as a manifestation of maladaptation. In the long run, it might trigger a series of externalizing and internalizing problem behaviors [40]. Externalizing problem behaviors, such as a cognitive attack, were specifically manifested as outward anger and hostility [63]. Anger was manifested as strong emotions, often in the form of frustration or annoyance. Hostility, however, was manifested as the bitterness, suspicion, and jealousy of others. Internalizing problem behaviors might induce depression and anxiety, resulting in a series of psychological problems [40]. Therefore, adopting negative coping imposed negative impacts on the transition shock of newly graduated nurses. Moreover, it was found that negative coping was associated with job burnout [17], which had been proven to be highly correlated to job satisfaction [64]. Kim and Yeo [34] explored the trend of newly graduated nurses' transition shock and job satisfaction within one year and believed that the clinical environment was the only factor that can affect the transition shock and job satisfaction. The results of this study showed that the coping style adopted by newly graduated nurses themselves when faced with transition shock was also a non-negligible moderator. This was consistent with the theory of organizational socialization for newly graduated nurses. In this theory, in addition to the positive support strategies from the organization, positive personal characteristics were also crucial in helping newly graduated nurses adapt to the new environment [46].

According to the COR theory, when individuals lose resources at work, they might experience stress in the form of burnout, depression, and physiological outcomes [51]. In such cases, to avoid further loss of resources, individuals automatically lowered their self-expectations of the role, maintained low motivation, and adopted the negative coping style of feedback avoidance [51]. Although employees might psychologically seek to leave work and work-related people to cope with emotional exhaustion, conversely, they might try to increase their acceptance in the organizational system to obtain social resources, especially support from colleagues [65]. Newly graduated nurses who had just entered the workforce had relatively fewer job resources. As a result, they might adopt negative coping to reduce the loss of resources when faced with transition shock. Meanwhile, they were also eager to get support and affirmation from colleagues in the department [66]. A study showed that [67], cooperation with the same preceptor during the internship and induction training could help newly graduated nurses rapidly establish a sense of belonging and adaptation, and help them make a smooth transition. It should be noted that in a study in the United States, researchers set up a new post, the “Nurse Retentionist,” which successfully reduced the turnover rate of newly graduated nurses [68]. Therefore, obtaining positive feedback from colleagues in the working environment was of vital importance for newly graduated nurses to reverse negative coping and actively face and overcome the transition shock.

In this study, on the one hand, how newly graduated nurses' transition shock influenced their intent to stay, i.e., the mediating effect of job satisfaction, was explained. On the other hand, the moderating effect of negative coping on the relationship between transition shock and job satisfaction was analyzed. Therefore, the results of this study were very important for nurse managers to develop more appropriate plans to help newly graduated nurses to go through the transition period smoothly and improve their intent to stay and retention rate.

## 5. Limitations

This study had some limitations. Firstly, the cross-sectional design of this study prevented us from drawing causal explanations. Therefore, the further longitudinal design needed to be taken into consideration. Secondly, the convenient sampling method was adopted in this study, and the samples were all from Shanghai, which was unfavorable to the generalization of samples. Therefore, the source of samples could be further expanded in future research. In addition, due to the use of online self-rating measurement, there might be response bias in our study. Future research could use multiple sources of data and types of indicators to improve it.

## 6. Conclusion

In this study, the influence of newly graduated nurses' transition shock on their intent to stay against the background of the COVID-19 pandemic was explored. Furthermore, the mediating effect of job satisfaction and the moderating effect of negative coping therein were found. At present, due to the COVID-19 pandemic, the contradiction between nurse shortage and the growing demand for clinical care has become more intense. These results are instructive for nursing managers to help newly graduated nurses successfully survive the transition period and improve their retention rate.

## 7. Implication for Nursing Management

The following recommendations were made based on the results of this study: (a) the level of the transition shock is an important indicator that reflects newly graduated nurses' transition state. It can further predict their job satisfaction and intent to stay. Therefore, nurse management should focus on adopting the corresponding measures to reduce the transition shock of newly graduated nurses. (b) It is necessary for newly graduated nurses to adopt positive coping to overcome the transition shock. This means that in the management of newly graduated nurses in the future, in addition to the existing transition programs, attention should also be paid to cultivating positive personal psychology. Positive psychological intervention (PPI) is worthy of consideration. It was proven in previous studies that mindfulness intervention (mindfulness-based program) could help newly graduated nurses smoothly go through the transition period [69]. (c) According to the COR theory, positive feedback from colleagues in the organizational environment is also necessary for newly graduated nurses faced with transition shock. In the study by Zhang, it was expounded that mentorship could provide newly graduated nurses with the necessary psychological and social support [70]. Therefore, allocating mentors to newly graduated nurses may be considered. In addition, adding new roles such as nurse retentionist or peer support may also be considered. Given the situation of the COVID-19 pandemic and the dependence of millennial generation nurses on electronics [13], it is also a good idea to build online support schemes.

To sum up, this study provides new insights that nurse managers can use to develop strategies for promoting newly graduated nurses' retention intention. Cultivating positive personal psychology and provide psychological and social support from mentors or peer colleagues are all worth-trying approaches to mitigate the transition shock of newly graduated nurses.

## Figures and Tables

**Table 1:** Results of comparative confirmatory analysis.

Measurement model	*χ* ^2^	d*f*	*χ* ^2^/d*f*	CFI	TLI	RMSEA	SRMR
Four-factor model (our hypothesized model)	1057.995	344	3.076	0.940	0.934	0.052	0.041
Three-factor model^a^	1901.146	347	5.479	0.870	0.859	0.077	0.061
Two-factor model^b^	4321.277	349	12.382	0.668	0.641	0.122	0.107
One-factor model^c^	4913.180	350	14.038	0.619	0.589	0.131	0.116

^a^This model combines transition shock and negative coping into one factor. ^b^This model combines transition shock, negative coping, and job satisfaction into one factor. ^c^This model combines all items into one factor.

**Table 2:** Results of JS as a mediator in the relationship between TS and STAY.

Criterion	Predictors	*R* ^2^	*B*	S.E.	*t*	*p*	95% CI	
JS	TS	0.354	−0.412	0.031	−13.370	<0.001	−0.478	−0.357

STAY	TS	0.545	−0.145	0.038	−3.829	<0.001	−0.216	−0.067
	JS		0.702	0.051	13.869	<0.001	0.614	0.810

TS: transition shock; JS: job satisfaction; STAY: intent to stay.

**Table 3:** Results of mediation model.

	*B*	S.E.	95% CI		Percentage (%)
Total effect	−0.434	0.037	−0.493	−0.357	100.00
Direct effect	−0.145	0.038	−0.206	−0.067	33.41
Indirect effect	−0.289	0.026	−0.338	−0.247	66.59

**Table 4:** Results of moderated-mediation model.

Criterion	Predictors	*R* ^2^	*B*	S.E.	*t*	*p*	95% CI	
JS	TS	0.390	−0.380	0.040	−9.439	<0.001	−0.467	−0.303
	NC		−0.055	0.030	−1.851	0.064	−0.110	0.010
	TS *∗*NC		−0.082	0.021	−3.969	<0.001	−0.125	−0.043

STAY	TS	0.545	−0.142	0.037	−3.799	<0.001	−0.211	−0.065
	JS		0.702	0.051	13.893	<0.001	0.616	0.816

TS: transition shock; JS: job satisfaction; STAY: intent to stay; NC: negative coping.

## Data Availability

The data that support the findings of this study are available from the corresponding authors upon reasonable request.

## Supplementary Materials

Supplementary Materials Supplementary Material Figure 1: mediating effects of job satisfaction. Supplementary Material Table 1: mean value, standard deviation, and correlation of all variables. Supplementary Material Table 2: results of the simple slope test.
